# A Case of NELL-1-Positive Membranous Nephropathy With Acute Kidney Injury Due to Bilateral Renal Vein Thrombosis

**DOI:** 10.7759/cureus.61230

**Published:** 2024-05-28

**Authors:** Pranjal Kashiv, Sunny Malde, Sushrut Gupta, Shubham Dubey, Kapil N Sejpal, Twinkle Pawar, Vrushali Mahajan, Prasad Gurjar, Amit Pasari, Manish Balwani

**Affiliations:** 1 Department of Nephrology, Jawaharlal Nehru Medical College, Wardha, IND; 2 Department of Medicine, Jawaharlal Nehru Medical College, Datta Meghe Institute of Medical Sciences (Deemed to be University), Wardha, IND; 3 Department of Pathology, Alexis Multispeciality Hospital, Nagpur, IND; 4 Department of Nephrology, Saraswati Kidney Care Center, Nagpur, IND

**Keywords:** thrombolysis, bilateral renal vein thrombosis, acute kidney injury, nell-1 antigen, primary membranous nephropathy

## Abstract

Membranous nephropathy (MN) is a significant cause of nephrotic syndrome in non-diabetic adults. It can be primary, attributed to autoantibodies targeting podocyte antigens, or secondary to various disorders. Although rare, nerve epidermal growth factor-like 1 (NELL-1)-associated MN presents diagnostic and management challenges. Thrombotic complications such as renal vein thrombosis (RVT) are recognized but less reported, especially in NELL-1-positive MN. We report a 43-year-old male with NELL-1-positive MN complicated by acute kidney injury (AKI) due to bilateral RVT, treated successfully with thrombolysis. Histopathological analysis confirmed MN with specific immunohistochemical staining for NELL-1. Treatment included immunosuppressive therapy and tailored anticoagulation. This case emphasizes recognizing thrombotic complications in MN, particularly in NELL-1-positive cases. Further research is needed to explore serum anti-NELL-1 antibodies as biomarkers and optimal anticoagulation strategies in MN patients at risk of thrombotic events to improve outcomes and guide personalized management.

## Introduction

Membranous nephropathy (MN) is a prevalent etiology of nephrotic syndrome in non-diabetic adults, making up approximately a third of biopsy diagnoses. MN represents about 20%-30% of nephrotic syndrome cases [1,2]. In adults, MN primarily presents as "primary," accounting for approximately 75%-80% of cases attributed to circulating autoantibodies targeting podocyte antigens [2,3]. Secondary MN accounts for about 20%-25% of cases in adults and is associated with various disorders, including infections such as syphilis and hepatitis B, malignancies, autoimmune diseases, allogeneic hematopoietic stem cell transplantation, and the use of certain drugs such as specific traditional medicines, nonsteroidal anti-inflammatory drugs (NSAIDs), and alpha-lipoic acid [3,4].

Primary MN is more commonly found in males over 40 years old. The causes of primary MN include phospholipase A2 receptor (PLA2R)-associated, nerve epidermal growth factor-like 1 (NELL-1)-associated, thrombospondin type 1 domain-containing 7A (THSD7A)-associated, and semaphorin 3B (Sema3B)-associated factors. Nerve epidermal growth factor-like 1 (NELL-1)-associated MN represents about 2.5% of all cases of MN. NELL-1 emerges as the second most common antigen in primary MN after PLA2R. NELL-1-associated MN exhibits a predominance of autoantibodies of immunoglobulin G1 (IgG1) [5].

In MN, the reported incidence comprises roughly 10%-30% for pulmonary embolism, 15% for deep vein thrombosis (DVT), and 25%-37% for renal vein thrombosis (RVT). RVT is a common finding in pediatric patients. Bilateral RVT causing acute kidney injury (AKI) is rare, especially in NELL-1-positive MN, with limited literature on its incidence and prevalence. Here, we present a case of NELL1-positive MN complicated by AKI due to bilateral RVT, successfully treated with thrombolysis. In this case report, we aim to highlight the potential treatable complication in nephrotic syndrome, which should be considered as a differential diagnosis of AKI in NELL1-positive MN patients [6,7].

## Case presentation

We present the case of a 43-year-old male referred to our center with abnormal kidney function test (KFT) results. He complained of bilateral pedal edema, frothy urine, shortness of breath on exertion for six months, and facial puffiness and abdominal distension for one month. He had been consuming alternative medicines for four months and had a known history of alcohol consumption for the past 10 years. Routine investigations were conducted, and the findings are outlined in Table 1.

**Table 1:** Changes in laboratory values during the clinical course of two hospitalizations RBCs: red blood cells, UPCR: urine protein creatinine ratio, ANA: antinuclear antibody

Investigations	On admission	On discharge	On readmission after 7 days	Post-thrombolysis	Reference range
Hemoglobin (gm/dL)	11.7	12	10	10.7	11-14
Creatinine (mg/dL)	3.8	2	6.8	2.5	0.66-1.25
Serum albumin (gm/dL)	1.6	1.7	1.7	2.1	3.4-5.4
Urine albumin	4+	NIL	3+	1+	<1+
Urine RBCs	Absent	Absent	7-8	Absent	Absent
UPCR (mg/mg)	2.13	-	-	-	<0.15
ANA	0.621	-	-	-	<0.01

Ultrasonography revealed the right kidney measuring 11.6 × 5.1 cm and the left kidney measuring 11.3 × 5.2 cm, with bilateral normal echotexture and maintained corticomedullary differentiation. Upon admission, his creatinine level increased to 3.8 mg/dL, hemoglobin was 11.7 gm/dL, platelet count was 2.21 lakh/mm^3^, serum albumin was 1.6 gm/dL, and urine albumin remained at 4+. Complement levels were normal. He was diagnosed with adult-onset nephrotic syndrome and subsequently underwent a renal biopsy after receiving informed consent.

**Figure 1:**
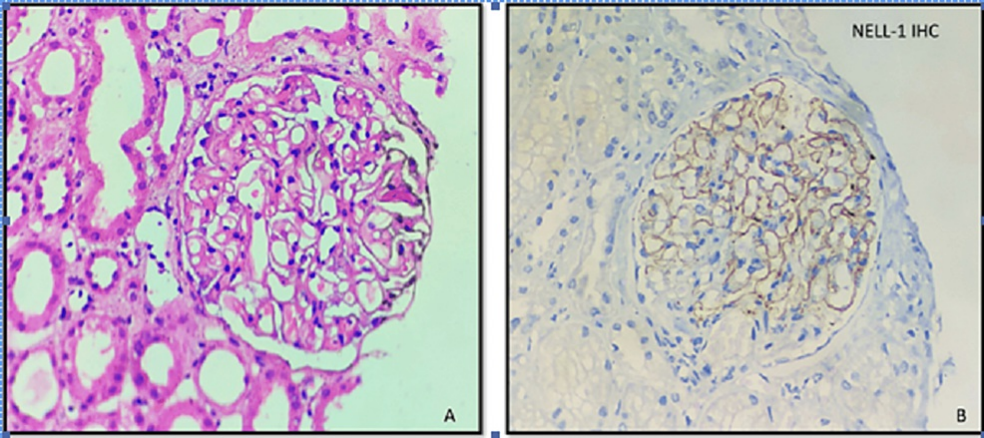
Renal biopsy findings A: H&E (40×): Microphotograph showing a glomerulus with uniform mild basement membrane thickening and rigidity with patent capillary lumina. The surrounding tubules show changes of acute tubular injury. B: NELL-1 IHC (40×): Microphotograph showing a glomerulus with peripheral, fine granular positivity of 2+ intensity along the glomerular basement membrane. H&E: hematoxylin and eosin, NELL-1: nerve epidermal growth factor-like 1, IHC: immunohistochemistry

Microscopic analysis of the kidney tissue revealed the following histopathological findings. On light microscopy, glomeruli displayed diffuse, uniform, mild basement membrane thickening with patent capillary lumina. The surrounding tubules showed changes in acute tubular injury. Immunofluorescence microscopy indicated the presence of diffuse and peripheral fine granular deposits of IgG with a 3+ intensity along the glomerular basement membrane (GBM). Immunohistochemistry (IHC) for PLA2R was negative, while positive staining for NELL-1 was noted. NELL-1 exhibited fine granular to pseudo-linear positivity with intensities ranging from 2+ to 3+ along the glomerular basement membrane (Figure 1).

The patient was diagnosed with NELL1-positive MN and was initiated on a modified Ponticelli regimen due to deranged glomerular filtration rate (GFR). The treatment commenced with pulse methylprednisolone at a dosage of 500 mg for three days, followed by oral prednisolone at a dosage of 1 mg/kg/day. Additionally, a computed tomography (CT) scan of the thorax and abdomen was conducted to exclude any associated malignancy, yielding negative results. Initially, the patient responded well to treatment, with creatinine levels decreasing to 2 mg/dL, leading to discharge. However, after seven days, he presented again with worsening symptoms and deranged kidney function, with an elevated creatinine level of 6.8 mg/dL.

**Figure 2:**
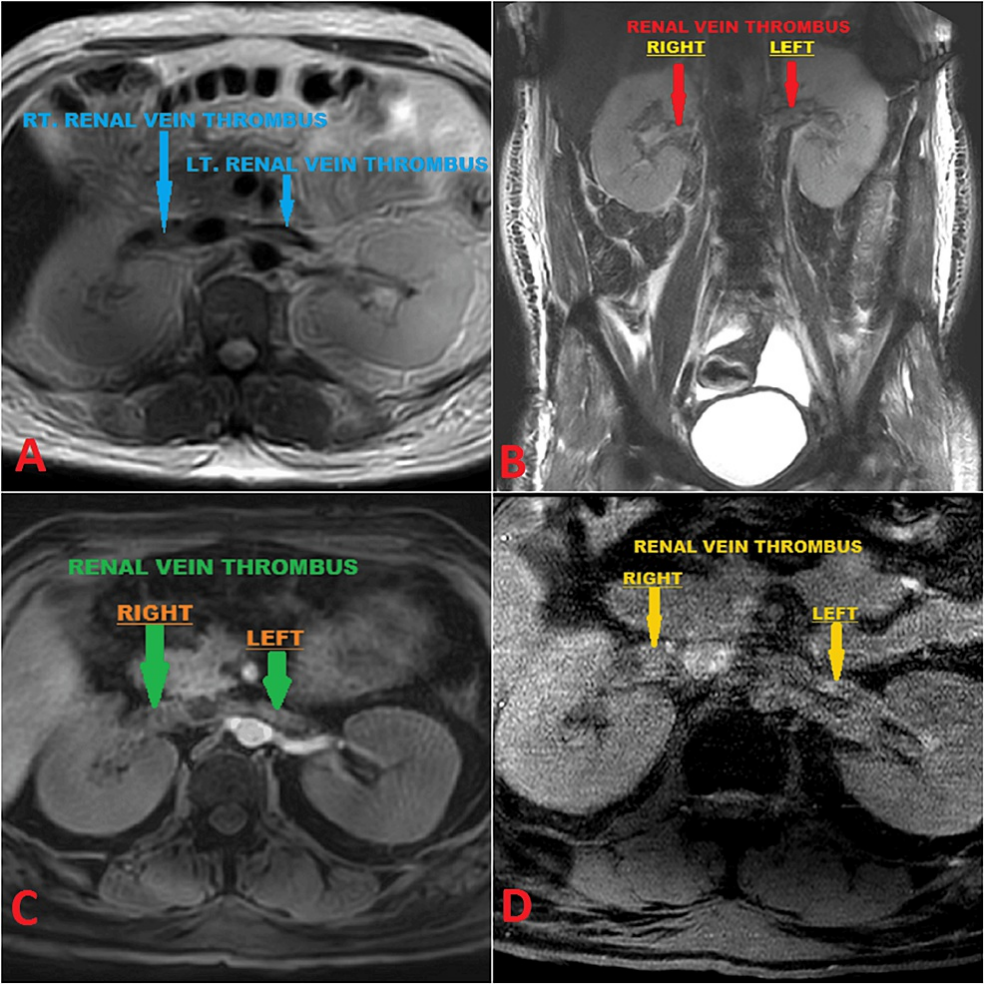
Bilateral renal vein thrombosis A: MR venography T2-weighted image depicting bilateral renal vein thrombus (blue arrows). B: MR venography T2-weighted image depicting bilateral renal vein thrombus (red arrows). C: 3D contrast-enhanced MR venography showing bilateral renal vein thrombus (green arrows). D: Partial thrombus in bilateral renal veins appearing as a filling defect in M2DI GS clear sequence (yellow arrows). MR: magnetic resonance

Further assessment for acute kidney injury (AKI) was initiated with an initial renal artery and vein Doppler examination, which returned normal results. However, due to persistent suspicion of thrombosis, subsequent magnetic resonance (MR) venography was conducted, confirming bilateral renal vein thrombosis (RVT) with partial extension from the hepatic part of the inferior vena cava (IVC) into both renal veins (Figure 2).

**Figure 3:**
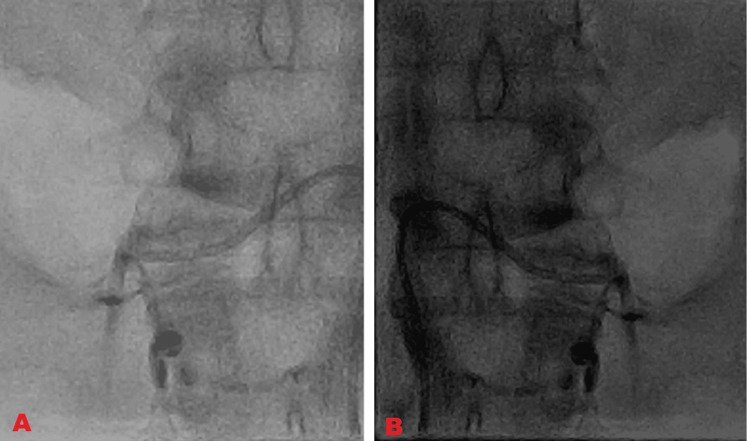
Post-thrombolysis A: Post-thrombolysis complete recanalization of the left renal vein. B: Post-thrombolysis near complete recanalization of the right renal vein.

Treatment involved heparin injection at 1,000 U/hour for 10 days and planned for the intervention of thrombolysis with injection alteplase (1.5 lakh units). Post-thrombolysis, complete recanalization of both renal veins occurred (Figure 3).

The patient continued injection heparin for an additional five days, subsequently transitioning to oral apixaban. Meanwhile, after one month of prednisolone therapy, the patient was transitioned to cyclophosphamide at a rate of 2.5 mg/kg/day. Eventually, the patient's condition improved, with creatinine levels reducing to 2.5 mg/day. Presently, the patient remains stable and is undergoing regular follow-up care.

## Discussion

MN is characterized by the accumulation of immune complexes along the glomerular basement membrane, often leading to nephrotic syndrome. Identified in 2009 and 2012, phospholipase A2 receptor (PLA2R) and thrombospondin type 1 domain-containing 7A (THSD7A) serve as target antigens in approximately 70% and 1%-5% of primary MN cases, respectively. Until recently, the target antigens in the remaining cases remained unknown. Advances in laser microdissection of kidney biopsy glomeruli and mass spectrometry revealed new antigens in PLA2R-negative MN, including NELL-1 [8].

In 2019, a study of 210 PLA2R-negative MN biopsies found 16% positivity for NELL-1. This exceeded twice the number of THSD7A-positive cases (15 out of 154 PLA2R-negative cases, 10%) [9]. In 2021, Caza et al. [10] found that 3.8% of MN cases negative for THSD7A and PLA2R were NELL-1 positive. Spain et al. [11] reported four biopsy-proven NELL-1-associated MN cases, with a fifth suspected case linked to lipoic acid supplementation, with remission achieved upon discontinuation. Additionally, Kudose et al. [12] described nine MN cases in hematopoietic stem cell transplant recipients, including two NELL-1-positive cases, raising considerations of associations with non-malignant conditions. Notably, in our case, the patient had no malignancy.

MN is pathologically characterized by diffuse thickening of the glomerular basement membrane (GBM) on light microscopy, "spikes" visible on silver stain, and diffuse, granular IgG and complement deposition on immunofluorescence. Additionally, subepithelial dense deposits are observed on electron microscopy. Notably, the histopathological characteristics of NELL1-associated MN stand out from other forms, exhibiting IgG1 subclass positivity, segmental to incomplete global IgG staining, and absence of staining for other immune reactants (IgA, IgM, and C1q) [5,6,8].

Thrombosis risk in nephrotic syndrome varies, with the highest in MN, followed by focal segmental glomerulonephritis (FSGS), and the lowest in IgA nephropathy (IgAN) [12]. In a study involving 1,313 patients, VTE frequency was highest in MN, correlating with proteinuria severity and inversely with albumin levels; serum albumin of <2.8 gm/dL had a 3.9 times increased risk, and <2.2 gm/dL had a 5.8 times increased risk, with each 1 gm/dL decrease in serum albumin corresponding to 2.13 times increased VTE risk [13,14]. The diagnosis and understanding of factors contributing to MN are crucial for timely intervention, preventing progression to end-stage renal disease (ESRD). Approximately one-third of MN patients develop ESRD. Prompt identification of MN is essential, even with limited laboratory abnormalities such as isolated hypoalbuminemia, due to the risks of ESRD and venous thromboembolism (VTE). Significant clinical findings include periorbital edema, hyperlipidemia, proteinuria, hypercoagulability, and hypoalbuminemia [15,16].

The management of acute RVT depends on whether the patient presents with AKI. Patients without AKI generally receive therapeutic anticoagulation, while those with AKI may undergo thrombolytic therapy with or without thrombectomy, followed by restorative anticoagulation maintenance [14,16]. Patients with undiagnosed nephrotic syndrome, especially in MN, may present initially with venous thromboembolism (VTE) or arterial thromboembolic (ATE) events. The current Kidney Disease: Improving Global Outcomes (KDIGO) guidelines suggest prophylactic anticoagulation in nephrotic syndrome patients, considering thromboembolic (TE) and bleeding risks [13-16]. Research suggests considering prophylactic anticoagulation for patients with severe hypoalbuminemia due to the hypercoagulable state in MN [13-17]. In our case, a patient with MN and bilateral RVT was initially treated with heparin but later underwent thrombolysis followed by apixaban due to inadequate response.

## Conclusions

MN is a prevalent cause of nephrotic syndrome in adults, with primary and secondary forms. NELL-1-associated MN is emerging as a distinctive entity. This case underscores the complex management of NELL-1-positive MN complicated by AKI from bilateral RVT. Swift diagnosis and intervention are crucial to prevent end-stage renal disease and thrombotic complications. Tailored management strategies should consider individual patient characteristics, including immunosuppression and anticoagulation. Further research on serum anti-NELL-1 antibodies as biomarkers and anticoagulation guidelines in MN patients is needed. A multidisciplinary approach involving nephrologists, hematologists, and interventional radiologists is vital for optimizing outcomes in such complex nephrotic syndromes.
